# Biosensors in Rehabilitation and Assistance Robotics

**DOI:** 10.3390/bios12110997

**Published:** 2022-11-10

**Authors:** Andres Ubeda, Gabriel J. Garcia, Carlos A. Jara, Vicente Morell

**Affiliations:** Human Robotics Group, University of Alicante, 03690 Alicante, Spain

Robotic developments in the field of rehabilitation and assistance have seen a significant increase in the last few years. The improvement of biosensing technologies provides robust ways of assessing the user’s neuromechanical behavior and mental states, which, when combined with robotic therapies, leads to critical improvements in motor or cognitive function recovery. Recent advances in bioelectrical signal processing and acquisition devices, in computer-vision techniques and machine-learning, and in the kinetic and dynamic analysis of movement have had a huge impact on the efficient development of the aforementioned robotic approaches.

This Special Issue is focused on breakthrough developments in the field of biosensors applied to rehabilitation and assistive robotics. The Special Issue has collected five outstanding papers covering different aspects of biosensing technologies and their use in different environments. What follows is a summary of the scope and main contributions of each of these papers, provided as a teaser for the interested reader.

The use of biosensors to monitor and assess progress during rehabilitation therapies is a key tool in many recent studies. For instance, the performance of a robotic ankle orthosis T-FLEX in combination with a lower-limb exoskeleton AGoRA has been evaluated using electromyographic (EMG) sensors to study muscular activity and using inertial sensors to study gait patterns [1]. Other parameters, such as metabolic costs and exerted forces, can be combined with EMG activity to evaluate the performance of these kinds of devices [2].

Another important application of biosensors in assistive technologies is their use as a way of inferring patients’ actual or intended motion to control external devices. A way of achieving this is by processing cortical activity with non-invasive electroencephalographic (EEG) devices. In [3], this approach is proposed to extract turning intention during human walking, showing the possibility of using this information to command robotic exoskeletons. EEG can also be useful to monitor cognitive performance during virtual reality-based interventions, as shown in [4]. In this case, another approach for motor rehabilitation is presented in [5], which proposes a sensorized arm wearable (ARMIA) that measures kinematic information through inertial sensors and muscular activity through EMG sensors. This device can be used both for active rehabilitation and to monitor physical performance.

## Data Availability

Not applicable.
